# Geraniin Inhibits the Entry of SARS-CoV-2 by Blocking the Interaction between Spike Protein RBD and Human ACE2 Receptor

**DOI:** 10.3390/ijms22168604

**Published:** 2021-08-10

**Authors:** Young Soo Kim, Hwan-Suck Chung, Sang Gyun Noh, Bonggi Lee, Hae Young Chung, Jang-Gi Choi

**Affiliations:** 1 Korean Medicine (KM) Application Center, Korea Institute of Oriental Medicine, Dong-gu, Daegu 41062, Korea; yskim527@kiom.re.kr (Y.S.K.); hschung@kiom.re.kr (H.-S.C.); 2 College of Pharmacy, Pusan National University, Busan 46241, Korea; rskrsk92@naver.com (S.G.N.); hyjung@pusan.ac.kr (H.Y.C.); 3 Department of Food Science and Nutrition, Pukyong National University, Nam-gu, Daeyeon Dong, Busan 608737, Korea; bong3257@pknu.ac.kr

**Keywords:** coronavirus disease 2019, severe acute respiratory syndrome coronavirus 2, geraniin, spike protein, hACE2 receptor

## Abstract

The coronavirus disease 2019 (COVID-19) pandemic is caused by severe acute respiratory syndrome coronavirus 2 (SARS-CoV-2). Despite the development of vaccines, the emergence of SARS-CoV-2 variants and the absence of effective therapeutics demand the continual investigation of COVID-19. Natural products containing active ingredients may be good therapeutic candidates. Here, we investigated the effectiveness of geraniin, the main ingredient in medical plants *Elaeocarpus sylvestris* var. *ellipticus* and *Nephelium lappaceum*, for treating COVID-19. The SARS-CoV-2 spike protein binds to the human angiotensin-converting enzyme 2 (hACE2) receptor to initiate virus entry into cells; viral entry may be an important target of COVID-19 therapeutics. Geraniin was found to effectively block the binding between the SARS-CoV-2 spike protein and hACE2 receptor in competitive enzyme-linked immunosorbent assay, suggesting that geraniin might inhibit the entry of SARS-CoV-2 into human epithelial cells. Geraniin also demonstrated a high affinity to both proteins despite a relatively lower equilibrium dissociation constant (*K*_D_) for the spike protein (0.63 μM) than hACE2 receptor (1.12 μM), according to biolayer interferometry-based analysis. *In silico* analysis indicated geraniin’s interaction with the residues functionally important in the binding between the two proteins. Thus, geraniin is a promising therapeutic agent for COVID-19 by blocking SARS-CoV-2’s entry into human cells.

## 1. Introduction

The coronavirus disease 2019 (COVID-19) pandemic, caused by an outbreak of a novel virus named severe acute respiratory syndrome coronavirus 2 (SARS-CoV-2) in December 2019, seriously threatens human health globally. SARS-CoV-2 genetically shares 79% and 51.8% identity with SARS-CoV and Middle East respiratory syndrome coronavirus (MERS-CoV), respectively, and causes respiratory infections in humans [1]. SARS-CoV-2 is more infectious, with a reproductive rate (R_0_) of 2.5 (range 1.8–3.6) than the H1N1 influenza A virus, SARS-CoV (2.0–3.0), MERS-CoV (0.9), and H1N1/09 virus (1.5) [2,3]. The high infectivity and fast mutation of SARS-CoV-2 have made the control of COVID-19 incredibly difficult.

After SARS-CoV-2’s spike protein binds to the human angiotensin-converting enzyme 2 (hACE2) receptor on the surface of a host epithelial cell, it is primed, or cleaved, by transmembrane serine protease 2 (TMPRSS2) before the virus enters the cells. Next, polyproteins are translated from the viral genome. After viral RNA synthesis and more protein production, the virus progenies are assembled and released from the cell [4].

To date, no effective therapeutic agents for COVID-19 have been developed. Although vaccines against SARS-CoV-2 have been developed, the emergence of SARS-CoV-2 variants requires continual research on the virus and the development of therapeutic agents and new vaccines. The development of various therapeutic agents targeting different stages in the life cycle of SARS-CoV-2 is underway [5,6]. The binding of the viral spike protein to hACE2 receptors is a good target because the inhibition of the interaction can abolish SARS-CoV-2’s entry into cells [7,8,9,10].

Since natural products are a good source of molecules for therapeutic development, many groups are investigating the therapeutic effects of natural products on COVID-19 [11,12,13,14,15,16,17]. Geraniin is a major component in medicinal plants, such as *Elaeocarpus sylvestris* var. *ellipticus* and *Nephelium lappaceum*, which are efficacious against various viruses, such as the influenza virus, hepatitis B virus, human enterovirus 71, herpes simplex virus type-1 and 2, and dengue virus type-2 [18,19,20,21,22]. However, the antiviral efficacy of geraniin against COVID-19 has not been examined, except for a study based on *in silico* docking simulation between geraniin and the spike protein receptor-binding domain (RBD) [11].

In this study, we investigated the potential of geraniin as a therapeutic agent for COVID-19 by targeting the entry of SARS-CoV-2 into cells. We evaluated geraniin’s effectiveness in blocking the binding of SARS-CoV-2 spike protein RBD to hACE2 receptors using ELISA (enzyme-linked immunosorbent assay) and biolayer interferometry. In addition, we analyzed the interactions between geraniin and the amino acid residues of the spike protein RBD or hACE2 receptor based on *in silico* docking simulations.

## 2. Results

### 2.1. Inhibition of Spike Protein RBD: hACE2 Receptor Interaction by Geraniin 

**Figure 1:**
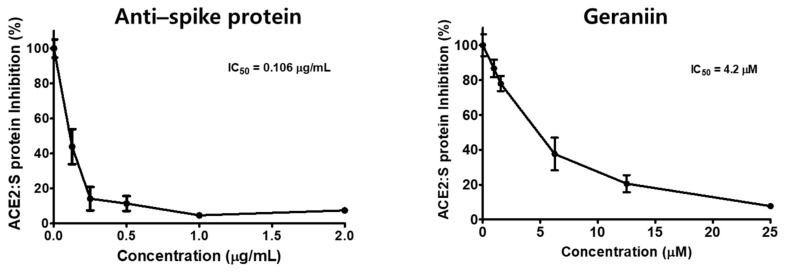
Inhibitory effect of geraniin on the binding of spike protein receptor-binding domain (RBD) to the human angiotensin-converting enzyme 2 (hACE2) receptor. Spike protein coated on a 96-well plate interacted with a pre-incubated mixture of the ACE2 receptor and 0, 0.78125, 1.5625, 6.25, 12.5, or 25 μM geraniin. The spike protein antibody was used as the positive control. The inhibition of the binding of the spike protein to the hACE2 receptor by geraniin was evaluated by measuring chemiluminescence.

We investigated the potential of geraniin in inhibiting the entry of SARS-CoV-2 into cells by evaluating the interaction between the spike protein RBD and the hACE2 receptor in the presence of geraniin using competitive ELISA (Figure 1). The spike S1 neutralizing antibody was used as the positive control. In the plate coated with spike protein RBD, the antibody dramatically blocked the binding between spike protein RBD and the hACE2 receptor with the IC_50_ value of 0.106 μg/mL. Geraniin effectively inhibited the interaction between spike protein RBD and the hACE2 receptor in a concentration-dependent manner, and the IC_50_ value of geraniin for this inhibition was 4.2 μM. These results suggested that geraniin could be developed to target the entry of SARS-CoV-2 into cells by blocking the interaction between the spike protein and the hACE2 receptor.

### 2.2. Affinity of Geraniin for Spike Protein RBD and hACE2 Receptor

**Figure 2:**
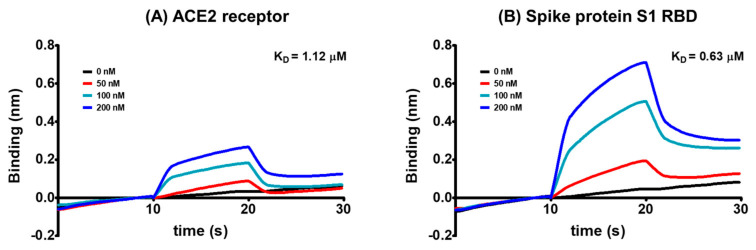
The global kinetic analysis of geraniin binding to biotinylated (**A**) hACE2 receptor and (**B**) spike protein S1 RBD immobilized on a streptavidin-coated sensor. The kinetics of geraniin for the spike protein or hACE2 receptor was measured by associating 0, 50, 100, and 200 nM of geraniin in PBS containing 1% DMSO with immobilized spike protein or hACE2 receptor and then dissociating in a blank solution.

We further investigated whether geraniin selectively bound to the spike protein S1 RBD and hACE2 receptor by evaluating geraniin’s affinity to either protein using a kinetic model based on the biolayer interferometry-based BLItz system. Geraniin demonstrated a high affinity to both spike protein S1 RBD and hACE2 receptors with the equilibrium dissociation constant (*K*_D_) of 0.63 × 10^−6^ and 1.12 × 10^−6^ M, respectively (Figure 2). The higher affinity of geraniin to S1 RBD than hACE2 receptor resulted more from the difference in dissociation constant (k_d_) values, at 2.57 × 10^−1^ s^−1^ for S1 RBD and 4.47 × 10^−1^ s^−1^ for the hACE2 receptor, than in association constant (k_a_) values, at 4.09 × 10^5^ M^−1^ s^−1^ for S1 RBD and 3.98 × 10^5^ M^−1^ s^−1^ for the hACE2 receptor.

### 2.3. Simulation and Pharmacophore Analysis of Geraniin with Spike Protein and hACE2 Receptor

**Figure 3:**
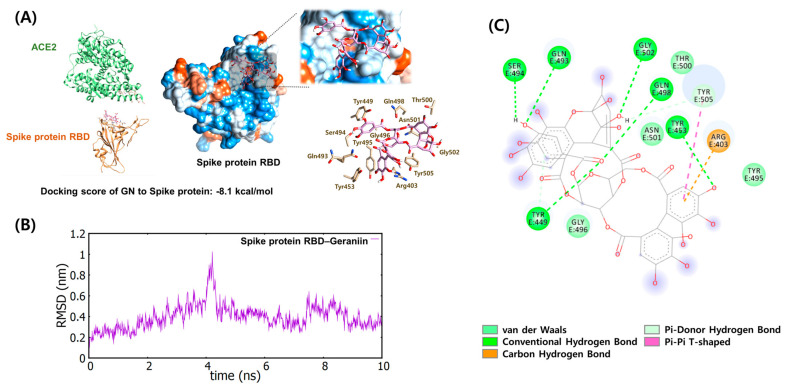
Protein-ligand docking simulation and analyses. (**A**) Docking simulation between geraniin and spike protein. Geraniin was docked onto the SARS-CoV-2 spike protein and hACE2 receptor derived from the spike protein/hACE2 receptor complex (PDB code 6M0J) using AutoDock Vina. (**B**) The molecular dynamics of spike protein–geraniin complex were performed using Gromacs 5.1.2. (**C**) The molecular interactions between geraniin and the spike protein were analyzed using BIOVIA Discovery Studio Visualizer.

We performed an *in silico* docking simulation to predict the amino acid residues in spike protein and hACE2 receptor that interacted with geraniin (Figure 3). The simulation demonstrated that geraniin might bind more stably to the spike protein with a lower docking score (∆G), at −8.1 kcal/mol, than to the hACE2 receptor at −7.0 kcal/mol. These data were consistent with the biolayer interferometry analysis. We further analyzed the conformational changes and stability of both docking complexes using molecular dynamic (MD) analysis. The spike protein–geraniin complex was stable, with a root mean square deviation (RMSD) of approximately 0.1 nm and 1.0 nm during simulations (Figure 3), whereas the hACE2–geraniin complex was partially unstable with an RMSD of approximately 0.1 nm and 10 nm (data not shown).

The pharmacophore analysis based on docking simulation showed that geraniin formed eight hydrogen bonds (Arg403, Tyr449, Tyr453, Gln493, Ser494, Gln498, Gly502, and Tyr505), four van der Waals interactions (Tyr495, Gly496, Thr500, and Asn501), and one pi-pi (Tyr505) interaction with the spike protein.

## 3. Discussion

Despite the recent development of several vaccines against SARS-CoV-2, the continuous emergence of SARS-CoV-2 variants and thne lack of effective therapeutic agents are still making the management of COVID-19 a serious challenge. Based on the life cycle of SARS-CoV-2, the development of COVID-19 therapeutics is mainly focused on viral entry into host cells and viral replication for new compounds and repurposed antiviral drugs [7,8,9,10].

During the early stages of the COVID-19 outbreak, many global biopharmaceutical companies tried to rapidly develop COVID-19 treatments through drug repurposing, and several drugs, such as remdesivir, favipiravir, chloroquine, and hydroxychloroquine, emerged as promising therapeutic candidates [8,23,24,25,26,27]. Although their administration clinically improved the recovery rate of patients, they were ineffective in reducing the mortality rate. Moreover, favipiravir was contraindicated due to the teratogenic and embryotoxic effects on animals [28,29,30]. Thus, the development of more effective therapeutic agents is crucial. In that respect, the investigation of natural products and their ingredients could be a good approach to discover new COVID-19 therapeutics with diverse backbones.

To date, virtual screening of natural and nature-derived products for activity against various targets in the different stages of the SARS-CoV-2 life cycle have suggested their potential as COVID-19 therapeutics [11,12,14,15,16,17]. However, the screening studies using actual SARS-CoV-2 particles are limited. Kanjanasirirat et al. investigated the efficacy of 122 natural products in Thailand in inhibiting the SARS-CoV-2 infection. They found that the culinary herb *Boesenbergia rotunda*, commonly cultivated in China and Southeast Asia, and its component panduratin A, significantly suppressed SARS-CoV-2 infectivity in Vero E6 cells (IC_50_: 3.62 μg/mL and 0.81 μM, respectively), and pre-treatment with panduratin A before SARS-CoV-2 entry inhibited viral infection (IC_50_: 5.30 μM) [13].

SARS-CoV-2 entry into cells occurs in three steps: (1) binding of SARS-CoV-2 to epithelial cells, (2) activation of spike protein by proteolysis, and (3) spike-mediated fusion [4,8,31]. Because SARS-CoV-2 binding results in interaction between the spike protein and hACE2 receptor, many studies are focusing on the development of small molecule- or peptide/antibody-based inhibitors that target the spike protein or hACE2 receptor [8,32]. Currently, several neutralizing antibodies against spike protein RBD have been developed and granted emergency use authorization for COVID-19 treatment by the U.S. Food and Drug Administration: regdanvimab (Celltrion Healthcare, Inc., Incheon, Korea), casirivimab and imdevimab cocktail (Regeneron Pharmaceuticals, Inc., Tarrytown, NY, USA), and bamlanivimab and etesevimab cocktail (Eli Lilly and Company, Indianapolis, IN, USA). Small molecule-based inhibitors have also been intensively studied because their use is preferential in terms of stability, pharmacokinetics, and administration [32,33,34].

**Figure 4:**
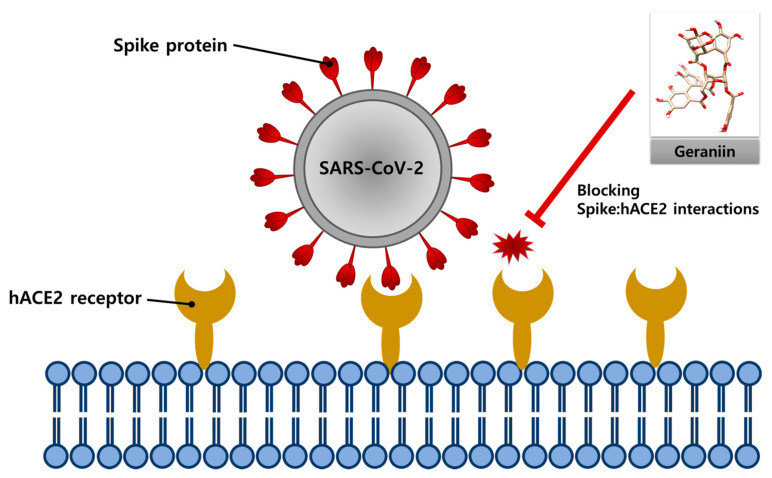
The diagram of the blockade of the SARS-CoV-2 spike protein: hACE2 receptor interactions by geraniin.

In this study, we investigated the potential of geraniin, a compound derived from natural products such as *Elaeocarpus sylvestris* var. *ellipticus* and *Nephelium lappaceum*, as a therapeutic agent for COVID-19. We discovered that geraniin blocked the interaction between the SARS-CoV-2 spike protein and the hACE2 receptor (Figure 4).

The efficacy of geraniin in hindering the interaction between the spike protein RBD and the hACE2 receptor was evaluated using competitive ELISA. The results showed that geraniin effectively prevented the binding of the spike protein RBD to the hACE2 receptor at a level comparable to the effect of the neutralizing antibody against the spike protein. The data suggest that geraniin can inhibit the entry of SARS-CoV-2 into human cells by targeting spike protein RBD or hACE2 receptor, thus preventing SARS-CoV-2 from binding to the cell surface.

Next, we investigated geraniin’s binding to the target proteins using a kinetic model based on biolayer interferometry. We determined the equilibrium dissociation constant (*K*_D_) of geraniin by measuring its association with and dissociation from the biotin-labeled spike protein S1 RBD and hACE2 receptor immobilized on streptavidin sensors. Geraniin exhibited a slightly higher affinity for spike protein S1 RBD than hACE2 receptor due to its dissociation constant (*k*_d_) more than its association constant (*k*_a_); however, the difference was insignificant. These data suggest that geraniin can interfere with the interaction between the spike protein and hACE2 receptor by targeting both proteins simultaneously, thereby preventing SARS-CoV-2’s entry into epithelial cells. However, hACE2 inhibition should be tightly controlled because hACE2 is involved in regulating cardiovascular processes as part of the renin–angiotensin–aldosterone system (RAAS) [35]. Thus, the spike protein might be a better target for the inhibition of SARS-CoV-2 entry, and further investigation regarding the effect of geraniin on the RAAS is required using in vivo experiments.

Based on the binding structure of the spike protein: hACE2 receptor complex revealed by X-ray crystallography [36], we predicted the binding of geraniin to the spike protein as well as the hACE2 receptor via *in silico* docking simulation. In the binding mode, geraniin binds more stably to the spike protein than to the hACE2 receptor, consistent with the results of the biolayer interferometry analysis. Additionally, MD simulations revealed that the spike protein–geraniin complex was very stable, with an RMSD of approximately 0.1–1.0 nm, whereas the hACE2–geraniin complex was partially unstable. Thus, pharmacophore analysis was only performed on the spike protein–geraniin complex.

The pharmacophore analysis based on the docking simulation showed that 12 amino acid residues of spike protein are mainly involved in their respective interaction with geraniin, and 11 of these amino acids are located in the receptor-binding motif (RBM, S438-Q506) of spike protein RBD [11]. Lan et al. suggest that 20 amino acid residues of the hACE2 receptor are in contact with 17 and 16 amino acid residues in the SARS-CoV-2 RBD and SARS-CoV RBD, respectively, indicating that SARS-CoV-2 and SARS-CoV RBDs have evolved convergently on the improved binding to hACE2 receptor. The SARS-CoV-2 spike protein shared eight identical (Y449, Y453, N487, Y489, G496, T500, G502, Y505) and five functionally similar (L455, F456, F486, Q493, N501) amino acids with the SARS-CoV spike protein. Y442, L472, N479, and T487 of the SARS-CoV spike protein, corresponding to L455, F486, Q493, and N501 in the SARS-CoV-2 spike protein, have been reported to play an essential role in the spike protein’s binding to the hACE2 receptor. In addition, geraniin was predicted to form hydrogen bonds (Q493, Y449, and Y505) and van der Waals interaction (N501) with the SARS-CoV-2 spike protein, thus hindering the interactions between the spike protein and the hACE2 receptor (K31, H34, E35, polar hydroxyl group D38 OD2, Q42 NE2, and E37 OE2; Y41, K353, G354, and D355. Arokiyaraj et al. also investigated the binding interactions between geraniin and spike protein RBD using *in silico* docking simulation [11]. Geraniin interacted with amino acid residues located slightly below the interface between spike protein RBD and hACE2 receptor T345, R346, and S349 (α1-β1) and L441, D442, and N450 (α4-β5 in RBM). They suggested that this strong binding of geraniin may have destabilized the spike protein RBD, resulting in the blockade of the interaction with hACE2 receptor. Thus, it will be interesting to further study the binding mode of the spike protein RBD–geraniin complex using X-ray crystallography to provide the hot spots for effective binding inhibition with hACE2 receptor.

The antibodies targeting SARS-CoV m396, S230, 80R, and CR3014 did not demonstrate significant binding and neutralizing activity to the SARS-CoV-2 RBD [37,38,39,40]. Lan et al. found that there were seven residue changes (A72, R403, T430, N439, Q498, P499, and N501 in SARS-CoV-2 RBD) among 21 epitope positions of m396, and 16 residue changes (N439, V445, G446, L452, L455, T470, G482, E484, G485, F486, F490, Q493, S494, Q498, P499, and N501) among 25 epitope positions of 80R. Therefore, these amino acid residues may provide important clues to the design of neutralizing agents for SARS-CoV-2. Pharmacophore analysis indicated that geraniin might interact with five (R403, Q493, S494, Q498, and N501) of these amino acids.

This study is the first to report, based on actual kinetic measurements, that the natural substance geraniin blocks the binding of SARS-CoV-2 to the hACE2 receptor. Our observation provides a structural clue for developing therapeutic agents for COVID-19 that inhibit SARS-CoV-2’s entry into human epithelial cells. At the same time, the *in silico* analysis in this study will help provide potential targets for developing small molecule-based therapeutic agents.

Further in vitro and in vivo studies on SARS-CoV-2 infection in the human epithelial cells and hACE2 transgenic mouse in the biosafety level-3 (BSL-3) laboratory are needed to develop geraniin into a SARS-CoV-2-binding inhibitor. Nevertheless, Biancatelli et al. observed that the treatment of the spike protein S1 subunit (S1SP) provoked the lung injury in the K18-hACE2 transgenic mice, which showed that the inhibition of the spike protein RBD–hACE2 receptor interactions by geraniin can be verified by simple treatment of S1SP in the K18-hACE2 transgenic mice without the BSL-3 laboratory [41]. The understanding gained from this study supports the significance and benefit of identifying and developing natural compounds for COVID-19 therapeutics.

## 4. Materials and Methods

### 4.1. Materials

Geraniin (ChemFaces, Wuhan, China) was purchased. The SARS-CoV-2 spike: ACE2 inhibitor screening assay kit, biotin-labeled recombinant protein ACE2 receptor, and spike protein S1 RBD (BPS Bioscience, San Diego, CA, USA) for biolayer interferometry were procured.

### 4.2. SARS-CoV-2 Spike/hACE2 Inhibitor Screening Assay

We tested the binding of geraniin to SARS-CoV-2 spike protein using the SARS-CoV-2 spike/hACE2 inhibitor screening assay kit. A 96-well plate was coated with 50 μL of 1 μg/mL SARS-CoV-2 spike protein in PBS overnight at 4 °C, washed with 100 μL of 1X immune buffer, blocked with 100 μL of 1X blocking buffer at room temperature for 1 h, and washed again. Then, 20 μL of 1X immune buffer was added to each well, followed by 10 μL of inhibitor solution containing the SARS-CoV-2 spike protein antibody (Active Motif, Carlsbad, CA, USA) or geraniin. The plates were incubated at room temperature for 1 h with slow shaking. Next, 20 μL of 2.5 μg/mL hACE2 inhibitor solution containing the geraniin or SARS-CoV-2 spike antibody was added for a 1-h incubation at room temperature with slow shaking. After 3 washes in 100 μL of 1X immune buffer, blocked with 100 μL of 1X blocking buffer per well at room temperature for 10 min, and washed again. Afterward, 100 μL of anti-His-HRP was added to each well, and the plates were incubated for 1 h. After incubation, the plates were washed 3 times with 1X immune buffer, blocked with 100 μL of 1X blocking buffer per well at room temperature for 10 min, and washed again. Relative chemiluminescence was measured using a GloMax-multi microplate reader (Promega, Madison, WI, USA).

### 4.3. Kinetic Analysis for the Binding between Geraniin and Spike Protein/ACE2 Receptor Based on Biolayer Interferometry

The binding affinities and kinetic constants of geraniin for the spike protein RBD and hACE2 receptor were evaluated using a BLItz system based on biolayer interferometry (Pall FortéBio, Fremont, CA, USA). After the pre-equilibration of the streptavidin BLI sensor (Pall FortéBio) in PBS buffer for 10 min, biotinylated spike protein S1 RBD and hACE2 receptor were fully loaded onto the sensors by soaking the sensor in 4 μL of 50 μg/mL spike protein RBD and hACE2 receptor solution. The geraniin solutions for kinetic analysis were prepared by diluting the stock solution in PBS with 1% DMSO to achieve 0, 50, 100, and 200 nM. 

The protein-immobilized sensor was first equilibrated in PBS buffer containing 1% DMSO for 20 s. Then, association in 4 μL of geraniin solution for 20 s was measured. Next, the dissociation in PBS containing 1% DMSO for 20 s was evaluated. The kinetic constants were calculated using BLItz Pro by fitting the association and dissociation data to a 1:1 model. The equilibrium dissociation constant, *K*_D_, was calculated as dissociation constant (*k*_d_)/association constant (*k*_a_).

### 4.4. In Silico Docking Simulation and Pharmacophore Analysis

SARS-CoV-2 spike protein and hACE2 receptor were retrieved from the Protein Data Bank (www.rcsb.org, PDB code: 6M0J) as receptors for docking simulations and geraniin (Chemspider ID 10270376) was used as a ligand. Prior to the docking simulations, receptor proteins and ligand were prepared by adding hydrogen atoms. With a minimum energy structure, geraniin was docked in a space with 25 × 40 × 30 dimension where the SARS-CoV-2 spike protein and hACE2 receptor were facing and interacting using AutoDock Vina integrated with UCSF Chimera 1.15. The binding affinity between geraniin and the spike protein/hACE2 receptor complex was presented as the lowest energy score in the docking simulation. The molecular interactions between geraniin and the spike protein/hACE2 receptor were analyzed and visualized by receptor–ligand interactions on a 2D diagram in BIOVIA Discovery Studio Visualizer.

### 4.5. Molecular Dynamic Analysis

The Gromacs 5.1.2 program was used to perform molecular dynamic simulations of the spike protein–geraniin and hACE2 receptor–geraniin complexes. Each protein molecular force field parameters were written in Gromos53a6 force field format, and geraniin molecular force field parameters were derived from Automated Topology Builder (ATB, https://atb.uq.edu.au/index.py, accessed date: 2 July 2021) written in Gromos54a7 force field format, which is converted into Gromacs format data. At the initial stage, energy minimization was performed using a 50,000-step steep descent method to obtain a stable conformation. After minimization, canonical ensembles (NVT) and isobar isothermal ensembles (NPT) were performed, respectively, with a constant temperature of 300 K for 100 ps for NVT, followed by a constant temperature of 300 K and a constant pressure of 1 atm per 100 ps for NPT. The production MD runs were then performed for 10 ns, keeping the temperature at 300 K and the pressure at 1 bar. The RMSD was calculated after the runs. The resulting graphics for the parameters were designed using the Gnuplot program.

### 4.6. Statistical Analysis

The data, presented as mean ± standard error of the mean, were analyzed using the GraphPad PRISM software (v5.02; GraphPad, San Diego, CA, USA).

## Data Availability

The data that support the findings of this study are available from the corresponding author upon reasonable request.
